# Supplementary material for: Aerogels Part 2. A Focus on the Less Patented and Marketed Airy Inorganic Networks Despite the Plethora of Possible Advanced Applications
Source: Int J Mol Sci. 2025 Nov 3;26(21):10696. doi: 10.3390/ijms262110696 (PMC12608421; doi:10.3390/ijms262110696)
Supplement: Supplementary file 1 [file ijms-26-10696-s001.zip › ijms-3910209-supplementary.pdf]

International Journal of Molecular Sciences

Supplementary Materials

Aerogels Part 2. A Focus on the Less Patented and Marketed Airy Inorganic Networks Despite the Plethora of Possible Advanced Applications

Silvana Alfei

Department of Pharmacy (DIFAR), University of Genoa, Viale Cembrano, 4, 16148 Genoa, Italy
Correspondence: alfei@difar.unige.it; Tel.: +39 010 355 2296 (S.A.)

Figures

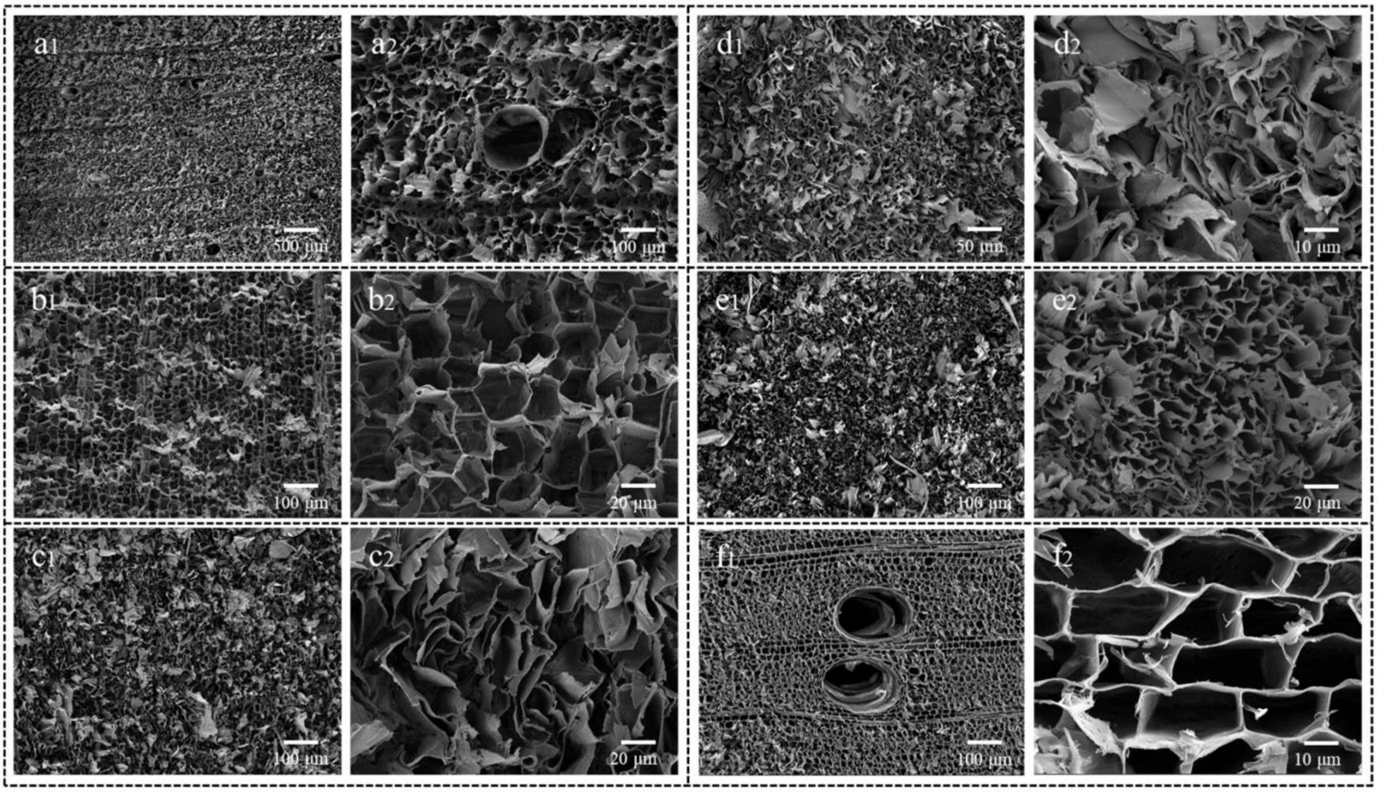

Figure S1. SEM images of the cross-sections of wood aerogels (WAGs): (1a), (2a) freeze-dried WAGs; (1b), (2b) supercritical CO_2-dried WAGs; (1c), (2c) vacuum-dried WAGs; (1d), (2d) oven-dried WAGs; (1e), (2e) naturally dried wood aerogel; (1f), (2f) original wood. This image has been reproduced by an open access article by Yin et al. [371], accessed on 02 September 2025 distributed under the terms and conditions of the Creative Commons Attribution (CC BY) license (https://creativecommons.org/licenses/by/4.0/, accessed on 02 September 2025).

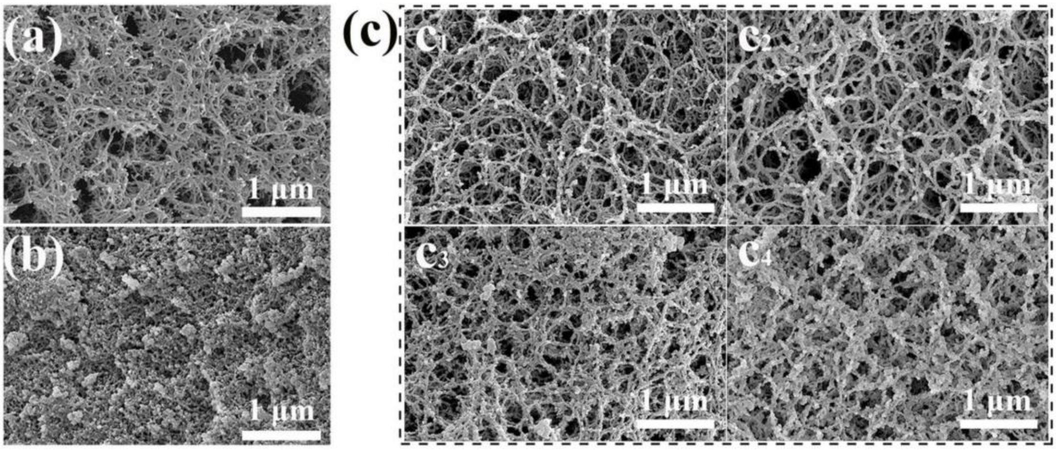

Figure S2. Microstructures of agarose AGs (AA-2) (a), silica AGs (SA-4) (b), and composite AGs (CAs) (c) by scanning electron microscopy (SEM) images, prepared by in situ sol-gel method. This image has been reproduced by an open access article by Yang et al. [372], accessed on 24 August 2025) distributed under the terms and conditions of the Creative Commons Attribution (CC BY) license (https://creativecommons.org/licenses/by/4.0/, accessed on 24 August 2025).

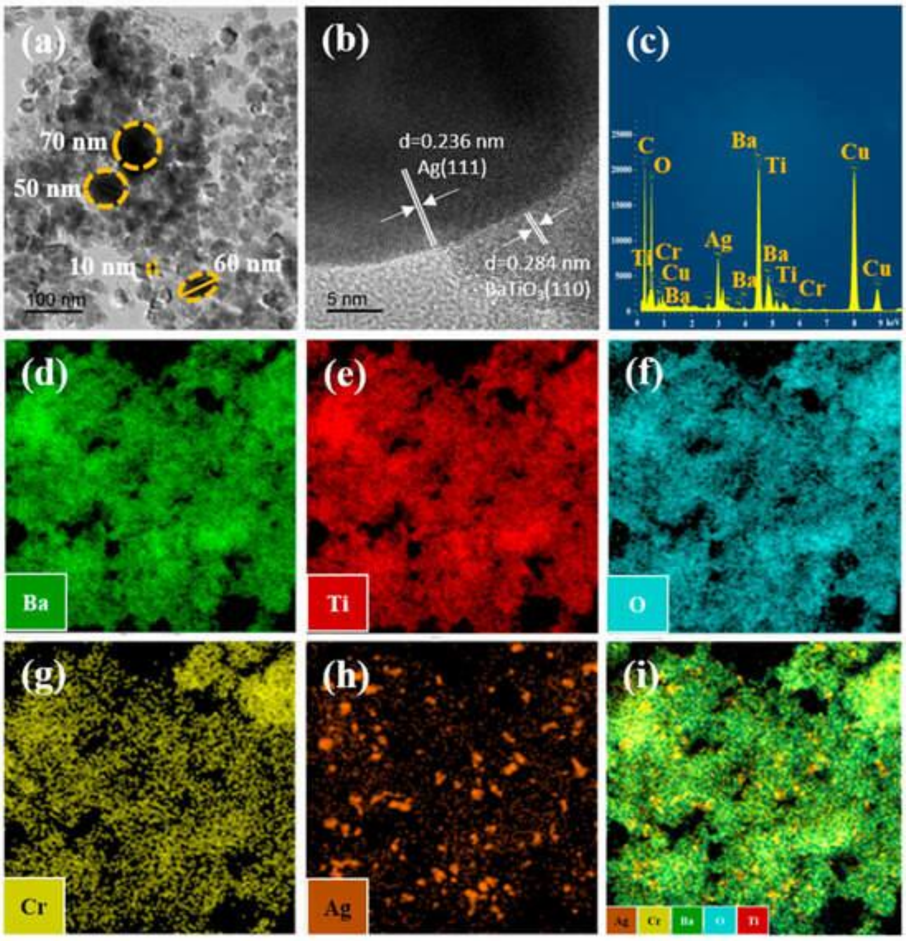

Figure S3. (a) TEM micrographs, (b) HRTEM micrographs, (c) STEM-EDX images, and (d–i) mapping images of Ag-modified Cr-doped $BaTiO_3$ (5% Ag/BTO-Cr010) AGs prepared by using a sol-co-gelation technique that involved two metallic alkoxides and a supercritical drying method, followed by Ag nanoparticles (Ag NPs) deposition. This image has been reproduced by an open access article

by Wu et al. [373], accessed on 24 August 2025) distributed under the terms and conditions of the Creative Commons Attribution (CC BY) license (https://creativecommons.org/licenses/by/4.0/, accessed on 24 August 2025).

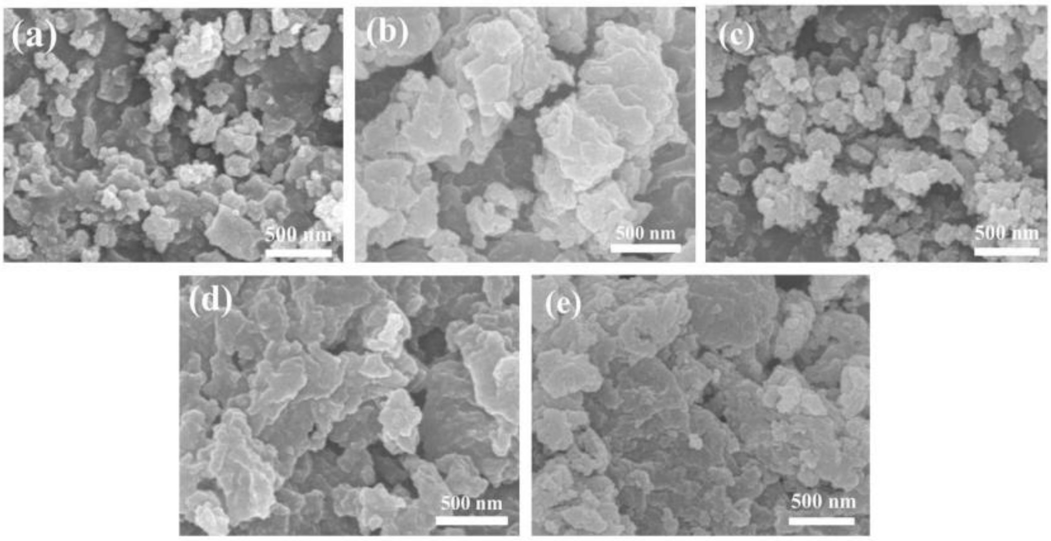

Figure S4. FE-SEM images of Zr–Mg mixed oxide AGs with Zr/Mg molar ratio of (a) 10/0, (b) 9/1, (c) 8/2, (d) 7/3, and (e) 6/4, prepared by the epoxide addition method. This image has been reproduced by an open access article by Lin et al. [374], accessed on 24 August 2025) distributed under the terms and conditions of the Creative Commons Attribution (CC BY) license (https://creativecommons.org/licenses/by/4.0/, accessed on 24 August 2025).

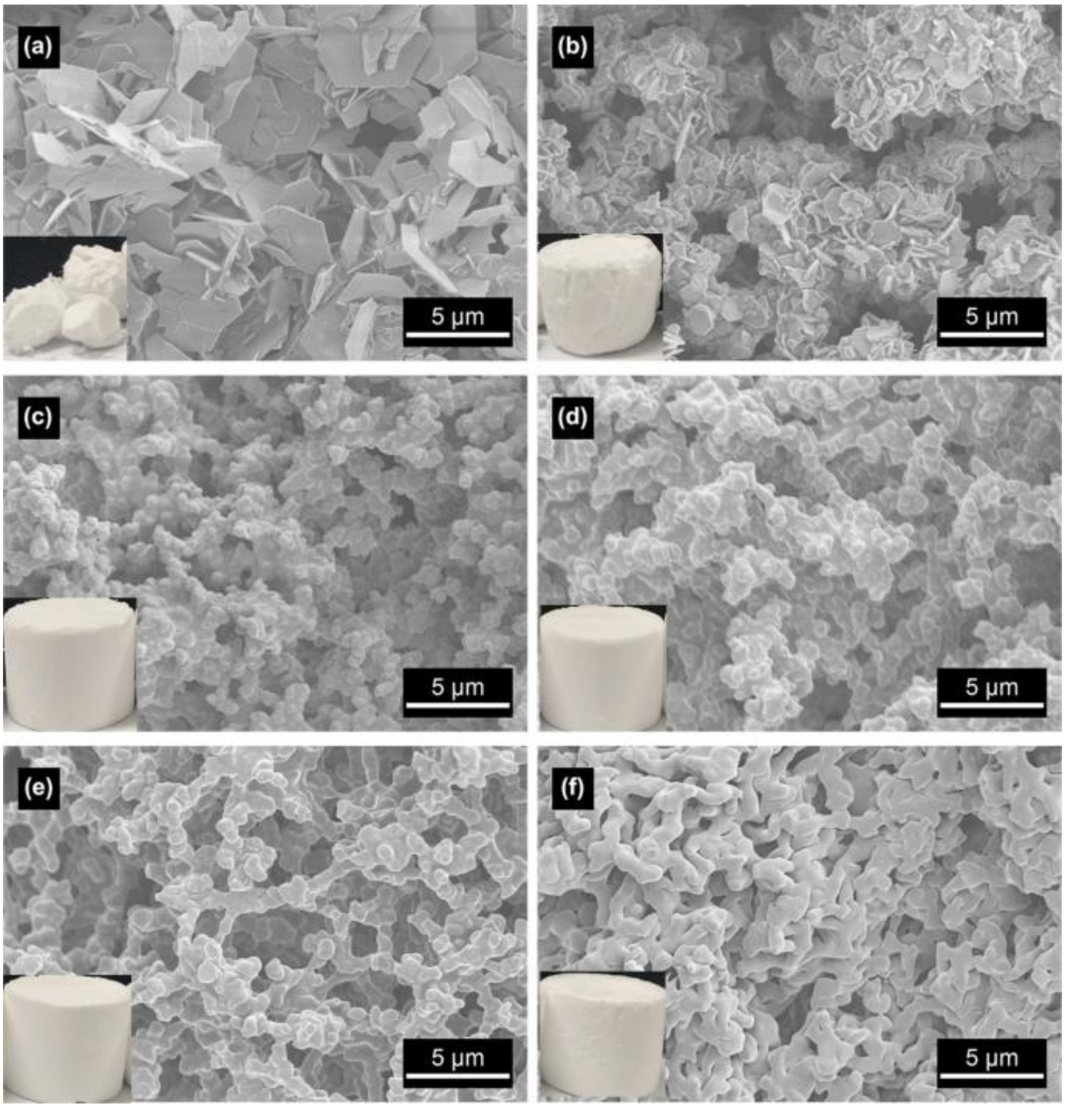

Figure S5. SEM images of xerogel samples with varied PAA amounts: (a) 0 g, (b) 0.8 g, (c) 1.6 g, (d) 2.4 g, (e) 3.2 g and (f) 4.0 g. This image has been reproduced by an open access article by Liu et al. [375], accessed on 31 August 2025) distributed under the terms and conditions of the Creative Commons Attribution (CC BY) license (https://creativecommons.org/licenses/by/4.0/, accessed on 31 August 2025).

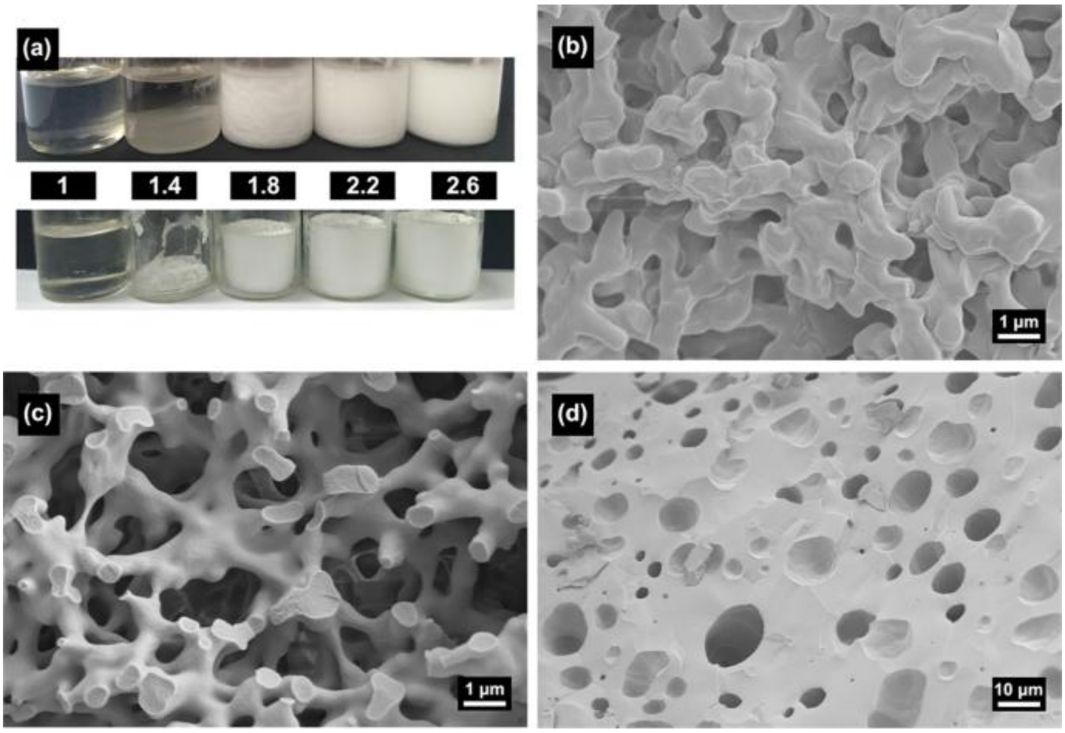

Figure S6. (**a**) Sol-gel transform in 30 min (up) and appearance of typical xerogel samples (down) and SEM images of xerogel samples with different PO contents: (b)1.8 mL, (c) 2.2 mL, (d) 2.6 mL. This image has been reproduced by an open access article by Liu et al. [375], accessed on 31 August 2025) distributed under the terms and conditions of the Creative Commons Attribution (CC BY) license (https://creativecommons.org/licenses/by/4.0/, accessed on 31 August 2025).

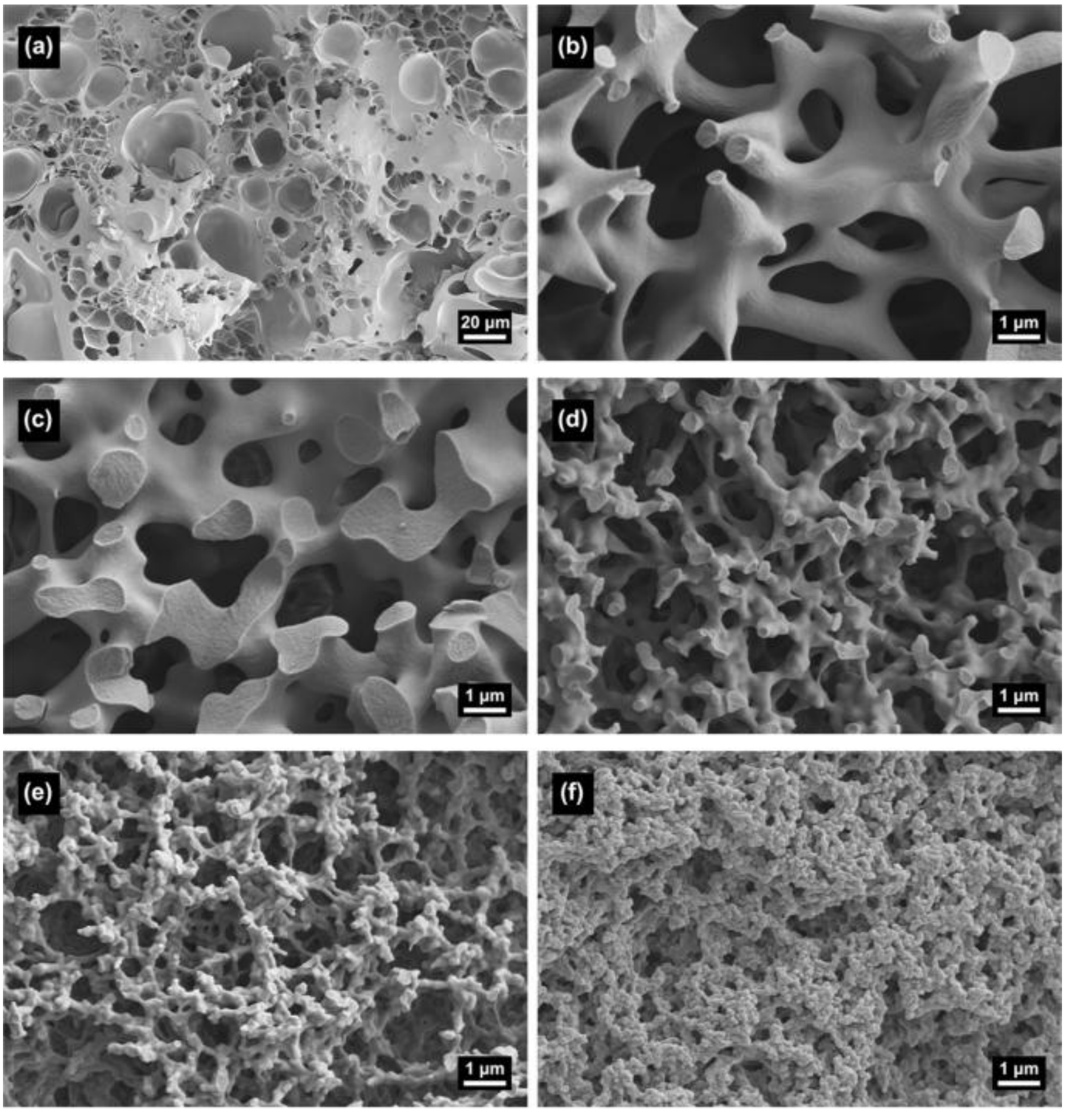

Figure S7. SEM images of xerogel samples with varied amounts of solvent, (a) W:G = 2:1.6, (b) W:G = 1.6:2.0, (c) W:G = 1.2:2.4, (d) W:G = 0.8:2.8, (e) W:G = 0.4:3.2 and (f) W:G = 0:3.6, respectively. W = water, G = glycerol. This image has been reproduced by an open access article by Liu et al. [375], accessed on 31 August 2025) distributed under the terms and conditions of the Creative Commons Attribution (CC BY) license (https://creativecommons.org/licenses/by/4.0/, accessed on 31 August 2025).

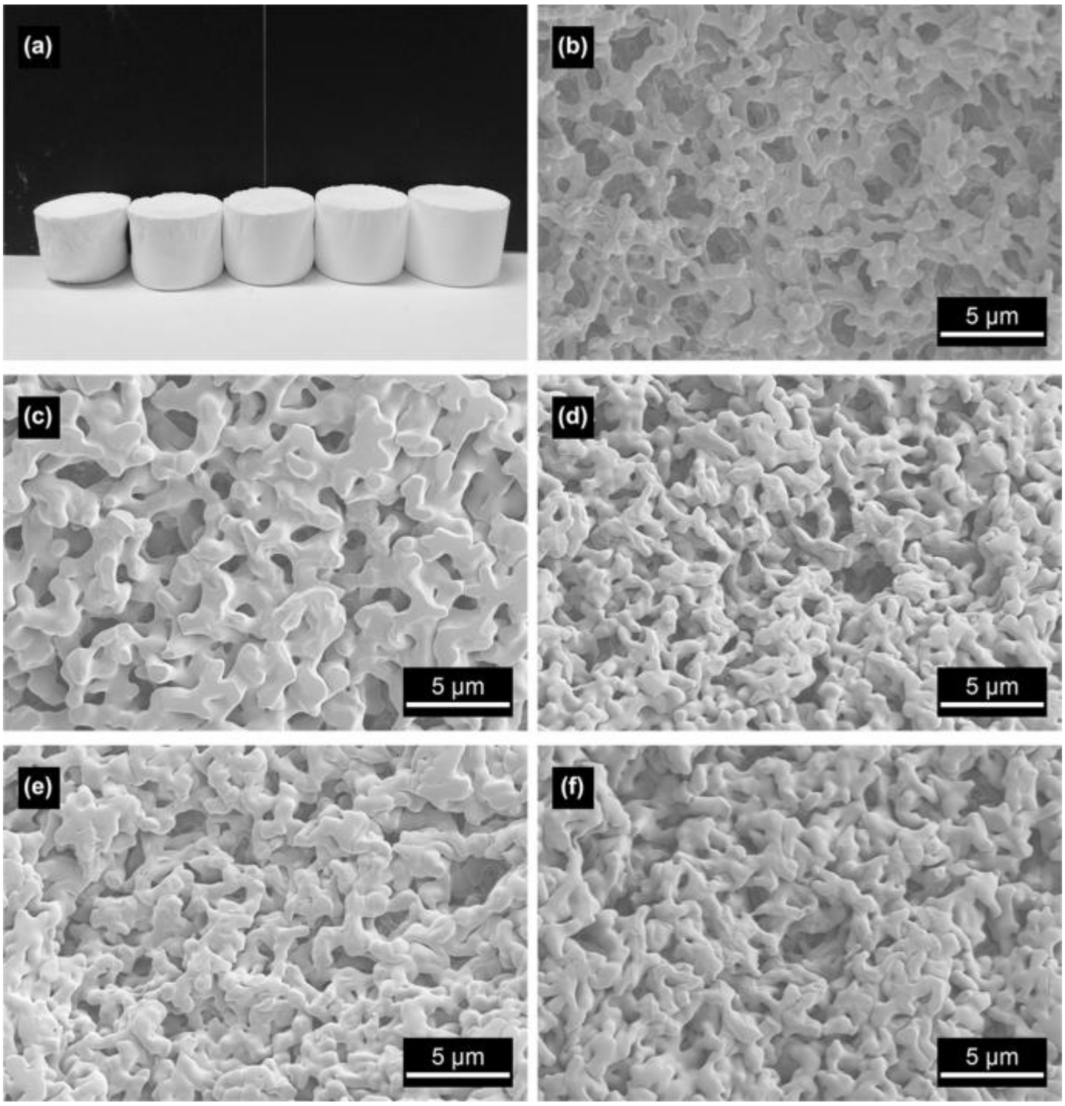

Figure S8. (a) Appearance of typical xerogel sample and SEM images of xerogel samples with varied amounts of precursors added; (b) 1.22 g, (c) 1.34 g, (d) 1.46 g, (e) 1.58 g and (f) 1.70 g, respectively. This image has been reproduced by an open access article by Liu et al. [375], accessed on 31 August 2025) distributed under the terms and conditions of the Creative Commons Attribution (CC BY) license (https://creativecommons.org/licenses/by/4.0/, accessed on 31 August 2025).

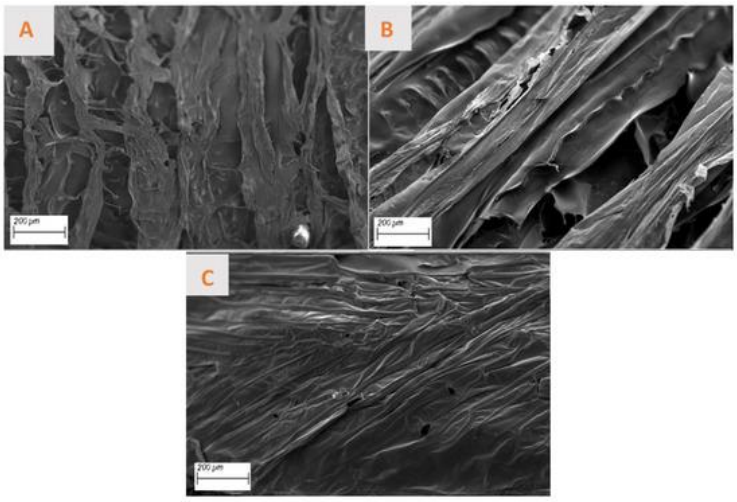

Figure S9. SEM micrographs of hybrid AGs prepared by combining collagen (C) and chitosan (CH) prepared without using nanoparticles (a, reference AG) and using chemical (b) and green (c) iron oxide nanoparticles dispersions, previously synthesized, as building blocks. This image has been reproduced by an open access article by Granados-Carrera et al. [376], accessed on 25 August 2025) distributed under the terms and conditions of the Creative Commons Attribution (CC BY) license (https://creativecommons.org/licenses/by/4.0/, accessed on 25 August 2025).

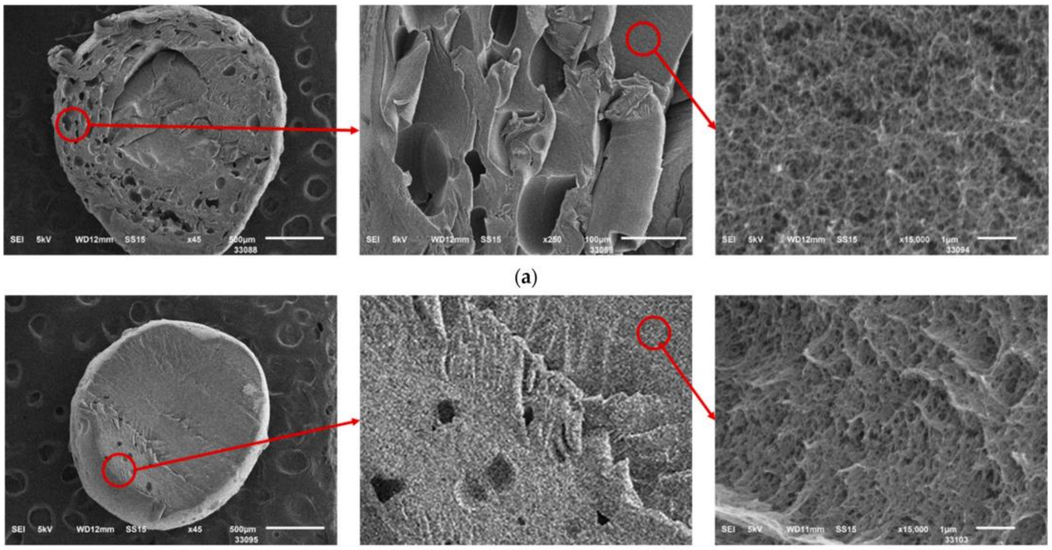

(**a**)

(**b**)

Figure S10. SEM images of the samples obtained by different methods: (**a**) soft-templating method using surfactants at a concentration of 0.5 wt.%; (**b**) hard-templating method using zein with a sodium alginate–zein ratio of 5:1. This image has been reproduced by an open access article by Menshutina et al. [377], accessed on 01 September 2025) distributed under the terms and conditions of the Creative Commons Attribution (CC BY) license (https://creativecommons.org/licenses/by/4.0/, accessed on 01 September 2025).

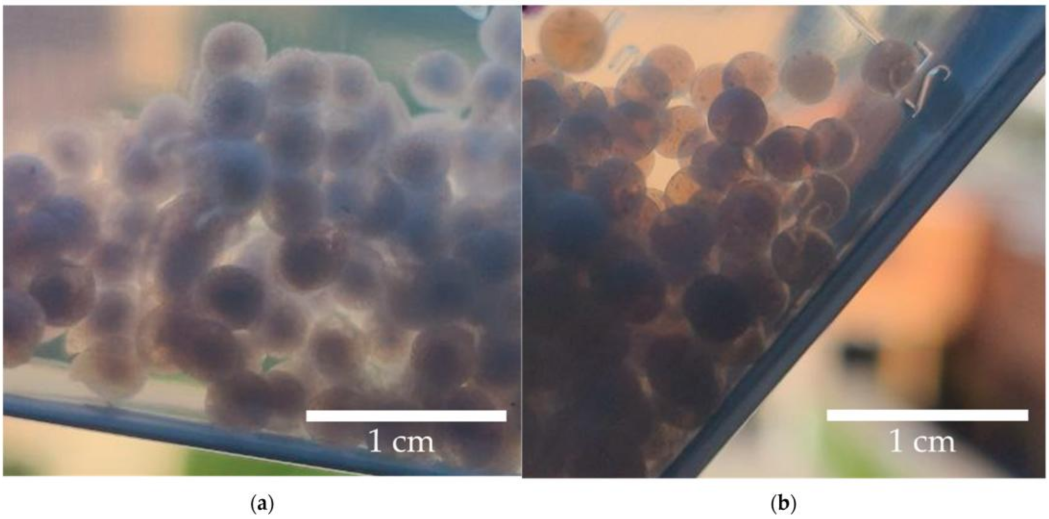

Figure S11. Appearance of samples obtained by (a) soft-templating methods using surfactants at a concentration of 0.5 wt.%; (b) hard-templating method using zein with a sodium alginate–zein ratio of 5:1. This image has been reproduced by an open access article by Menshutina et al. [377], accessed on 01 September 2025) distributed under the terms and conditions of the Creative Commons Attribution (CC BY) license (https://creativecommons.org/licenses/by/4.0/, accessed on 01 September 2025).

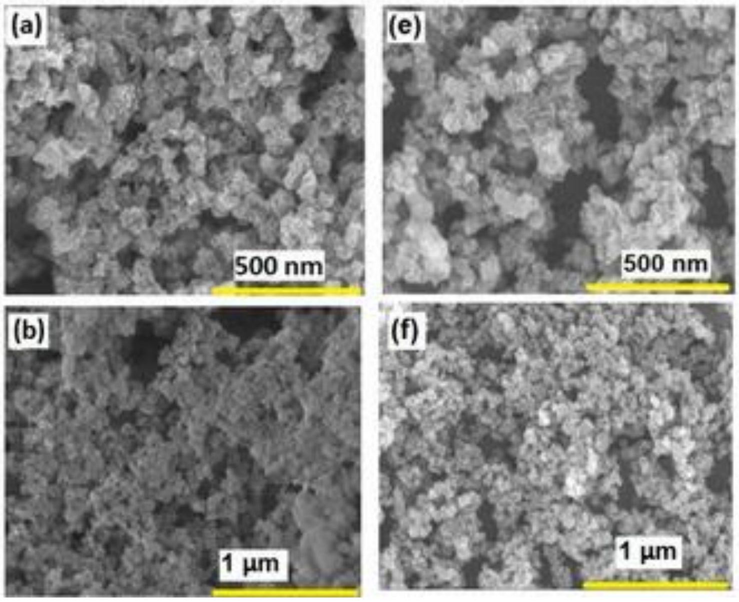

Figure S12. FE-SEM images of α-$Ni(OH)_2$ AGs prepared by using a two-step sol-gel method followed by a freeze-drying technique (a,b) and of the NiO/Ni aerogels obtained by the prepared α-$Ni(OH)_2$ aerogels through annelation at 400 °C (e,f). This image has been reproduced by an open access article by Ramkumar et al. [378], accessed on 25 August 2025) distributed under the terms and conditions of the Creative Commons Attribution (CC BY) license (https://creativecommons.org/licenses/by/4.0/, accessed on 24 August 2025).

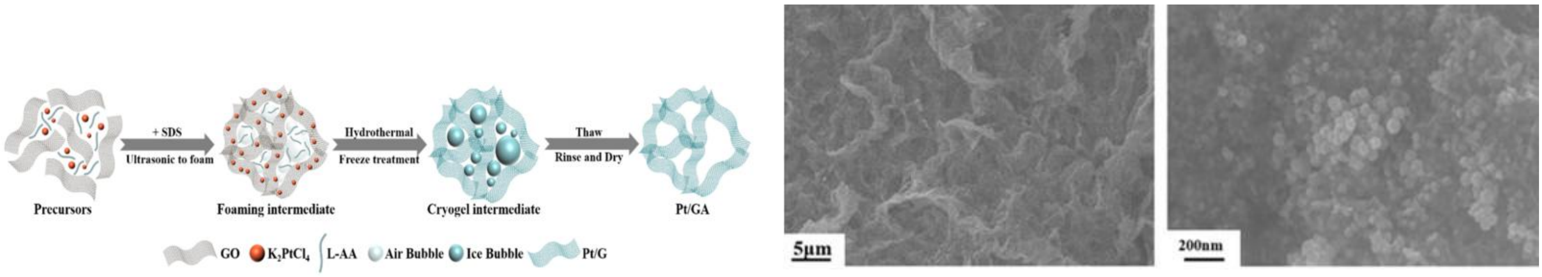

(a) (b)

Figure S13. Scheme of the one-step hydrothermal self-assembly method used to prepare a platinum NPs supported graphene AG (Pt/3DGA) catalyst (Figure S13a); SEM images of the Pt/3DGA at different magnifications (Figure S13b). This image has been reproduced by an open access article by Wo et al. [379], accessed on 31 August 2025) distributed under the terms and conditions of the Creative Commons Attribution (CC BY) license (https://creativecommons.org/licenses/by/4.0/, accessed on 31 August 2025).

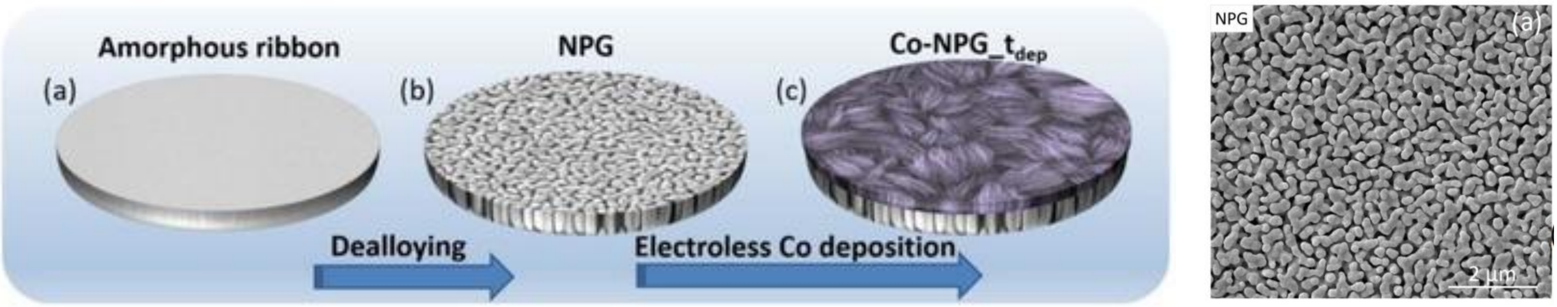

(A) (B)

Figure S14. (A) Schematic preparation of cobalt porous gold nanoparticles (Co-NPG_t_{dep}) (**a**) Synthesis of the amorphous ribbon through melt spinning process; (**b**) dealloying process to obtain NPG; (**c**) electroless Co deposition onto NPG at selected times. (B) SE-SEM image of the porous NPG obtained by dealloying of the amorphous precursor. This image has been reproduced by an open access article by Barrera et al. [380], accessed on 24 August 2025) distributed under the terms and conditions of the Creative Commons Attribution (CC BY) license (https://creativecommons.org/licenses/by/4.0/, accessed on 24 August 2025).

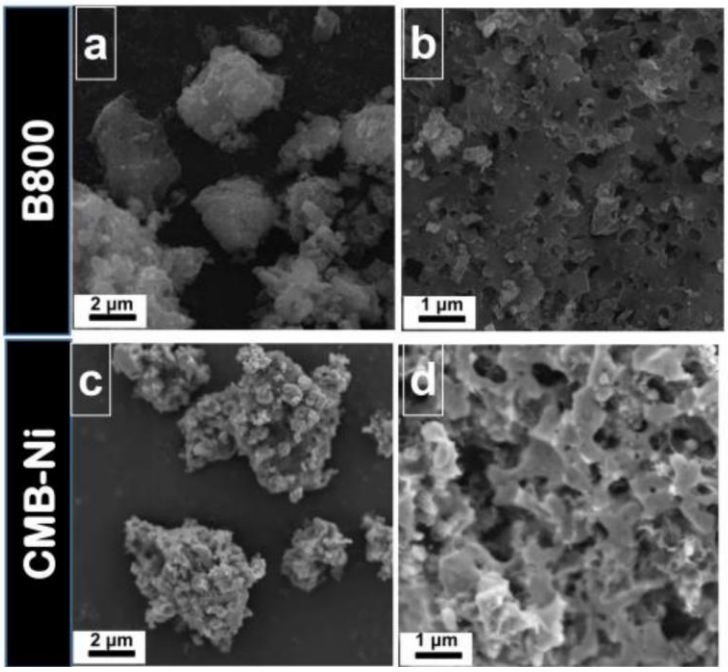

Figure S15. SEM images of biogenetic MnO/C (CMB), namely B800 (Figure S15a, b) and MnO/C/NiO (CMB-Ni) (Figure S15c, d) porous composites. They were obtained for combustion in a tube furnace at 800 °C (4 h, ramp of 5 °C min−1 under an Ar atmosphere) of the MnO_2/bacteria (BMB) and of the MnO_2/bacteria/Ni (BMB-Ni) porous composites prepared by a bio-templated method based on *Pseudomonas putida* cell-surface display technology. This image has been reproduced by an open access article by Liu et al. [381], accessed on 25 August 2025) distributed under the terms and conditions of the Creative Commons Attribution (CC BY) license (https://creativecommons.org/licenses/by/4.0/, accessed on 25 August 2025).

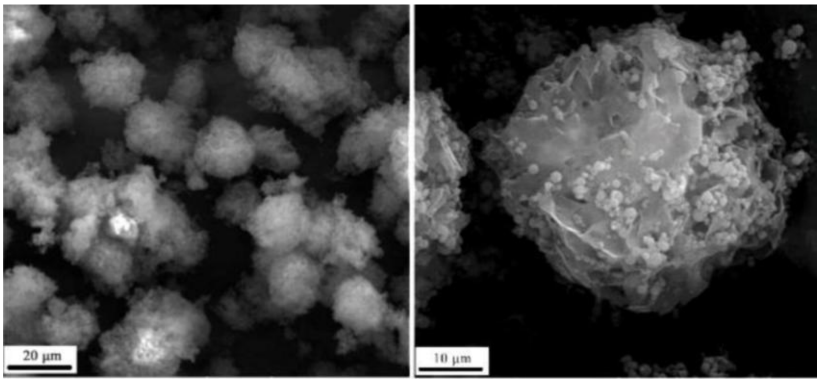

Figure S16. SEM images of biogenic MnO_2/bacteria (BMB) porous composites prepared by a bio-templated method based on *Pseudomonas putida* cell-surface display technology. This image has been reproduced by an open access article by Liu et al. [381], accessed on 25 August 2025) distributed under the terms and conditions of the Creative Commons Attribution (CC BY) license (https://creativecommons.org/licenses/by/4.0/, accessed on 25 August 2025).

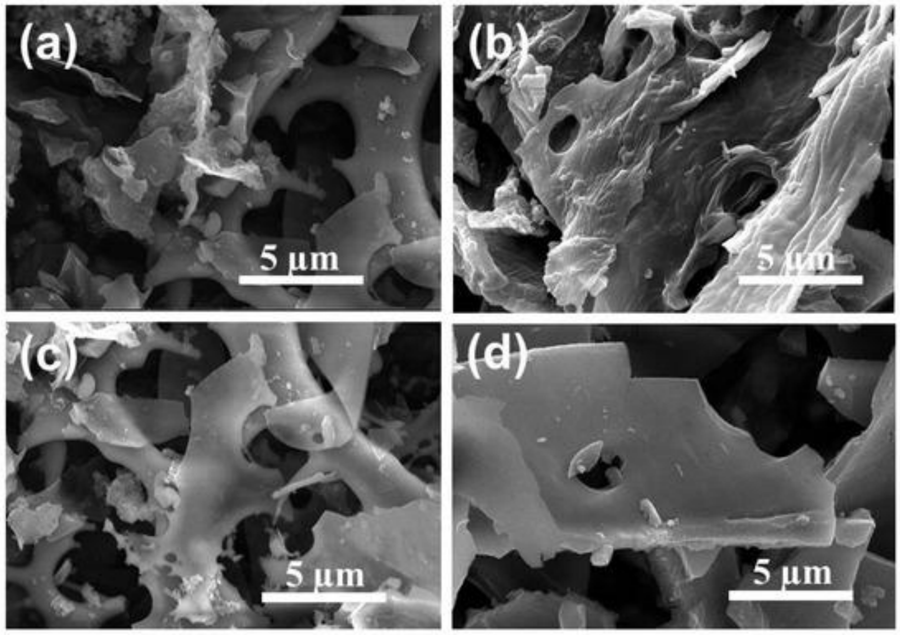

Figure S17. SEM images of biomass-derived porous carbon materials with a good balance between high specific surface area and mesopore volume prepared via a molten chloride salt templating technique and successive KOH activation (MHPC-700, MHPC-800, and MHPC-900) (a, b, c). SEM image of the carbon sample NHPC-700 pre-carbonized in nitrogen atmosphere without molten salt (d). This image has been reproduced by an open access article by Wang et al. [382], accessed on 25 August 2025) distributed under the terms and conditions of the Creative Commons Attribution (CC BY) license (https://creativecommons.org/licenses/by/4.0/, accessed on 25 August 2025).

Disclaimer/Publisher's Note: The statements, opinions and data contained in all publications are solely those of the individual author(s) and contributor(s) and not of MDPI and/or the editor(s). MDPI and/or the editor(s) disclaim responsibility for any injury to people or property resulting from any ideas, methods, instructions or products referred to in the content.
